# Electroacupuncture Relieves Visceral Hypersensitivity by Inactivating Protease-Activated Receptor 2 in a Rat Model of Postinfectious Irritable Bowel Syndrome

**DOI:** 10.1155/2018/7048584

**Published:** 2018-10-18

**Authors:** Wanli Xu, Mengqian Yuan, Xiaoliang Wu, Hao Geng, Lu Chen, Junling Zhou, Yafang Song, Lixia Pei, Jianhua Sun

**Affiliations:** ^1^Affiliated Hospital of Nanjing University of Traditional Chinese Medicine, Jiangsu, China; ^2^Jiangsu Province Hospital of Traditional Chinese Medicine, Jiangsu, China

## Abstract

**Background:**

The role of protease-activated receptor 2 (PAR2) in the analgesic effect of electroacupuncture (EA) on visceral hypersensitivity (VH) in postinfectious irritable bowel syndrome (PI-IBS) has yet to be elucidated.

**Aim:**

In this study, we investigated the molecular mechanisms underlying the analgesic effect of EA in a rat model of PI-IBS.

**Methods:**

Visceral hypersensitivity was evaluated by the abdominal withdrawal reflex test before and after administration of the PAR2 agonist, PAR2-AP, and/or EA. The protein expression and mRNA levels of PAR2, CGRP, SP, and TPSP in colon tissues were measured by immunofluorescence, western blot, and RT-PCR.

**Results:**

We found that EA could alleviate VH and significantly decrease protein and mRNA levels of PAR2, TPSP, CGRP, and SP in PI-IBS rats. The analgesic effect of EA on VH was slightly reduced in the presence of PAR2-AP.

**Conclusions:**

These results suggest that EA alleviates VH symptoms through downregulation of the levels of the TPSP/PAR2/SP/CGRP signaling axis in colon tissues in PI-IBS rats. Together, our data suggests that PAR2 plays a critical role in the analgesic effect of EA on VH in PI-IBS.

## 1. Introduction

Irritable bowel syndrome (IBS) is a common, chronic, functional gastrointestinal disorder, characterized by visceral hypersensitivity (VH) or abdominal discomfort, as well as altered bowel habits, without obvious anatomical or biochemical abnormalities. Infectious enteritis was identified as a risk factor for the development of IBS in the 1960s, and there is growing evidence that IBS is often followed by acute gastroenteritis, a condition termed “postinfectious irritable bowel syndrome (PI-IBS)” [1–4].

Augmentation of visceral afferent sensation (nociception) may be a major cause of VH in PI-IBS. VH is associated with altered bowel movements, increased mucosal secretion, and a lowered sensory threshold to colorectal distention in humans and animals. It has been hypothesized that VH is caused by triggering events, such as injury and inflammation, with the ensuing release of various neurogenic factors provoking sustained depolarization of the primary afferent nerve terminals, thereby exciting the spinal and supraspinal neurons [5]. A major obstacle to the effective treatment of IBS is that the mediators of symptoms such as abdominal pain and their mechanisms of action are unknown.

It was found that VH may be the result of immune-neuronal overactivation, during which PAR2 activates and transmits painful stimuli, leading to neuro-hypersensitivity. Colon tissue supernatants from IBS-D patients can directly activate the nociceptive perception pathway and induce pain when applied to rat, and this can be blocked by PAR2 receptor antagonism or gene knockout [6, 7]. PAR-2 at nerve endings can be cleaved by TPSP derived from immune cell activation, a main mediator of PAR-2, then sensitize transient receptor potential, and cause the release of neuropeptides CGRP and SP that transmit pain signals. Previous studies have suggested a crucial role for protease-activated receptor 2 (PAR2), which is activated by proteases in the colonic tissues of IBS patients, as a mediator contributing to hypersensitivity symptoms [8–10].

Although many years of intensive research have shown that electroacupuncture (EA) can alleviate PI-IBS-associated VH [11, 12], our understanding of the molecular signaling mechanisms affected by EA remains incomplete. In this study, we sought to determine whether EA could regulate mediators released by colonic tissues that signal to visceral nociceptive neurons and cause VH symptoms in PI-IBS rats. The acupoints ST36 and BL25 were selected for stimulation, both of which not only are the main acupoints for treating gastrointestinal diseases in traditional Chinese medicine theory, but also proved to be related to the periodic dominance of gastrointestinal nerves. We focused our attention on PAR2, since it has been shown to cause VH symptoms in PI-IBS rats, and we also investigated tryptase (TPSP), which is upstream of PAR2, as well as substance P (SP) and calcitonin gene-related peptide (CGRP) which are regarded as secondary neurotransmitters in colon tissues. Overall, our data reveal that EA alleviates VH symptoms via inactivation of the TPSP/PAR2/SP/CGRP signaling axis in colon tissues in PI-IBS rats. Furthermore, we demonstrate the critical role of PAR2 in the analgesic effect of EA on VH in PI-IBS rats.

## 2. Materials and Methods

### 2.1. Study Design

**Figure 1:**
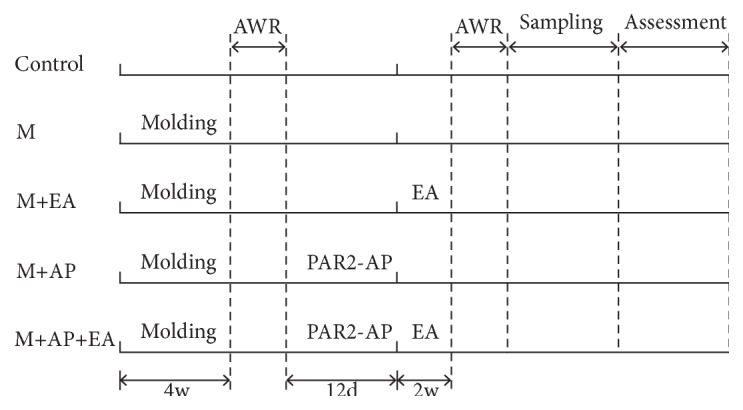
**Experimental procedure**. Intervention timeline for the control (C) group, model (M) group, M + electroacupuncture (EA) group, M + PAR2-agonist peptide (AP) group, and M + AP + EA group. The M group was administered with TNBS; the M + EA group was administered with both TNBS and EA; the M + AP group was administered with both TNBS and PAR2-AP; the M+AP+EA group was administered with TNBS, PAR2-AP, and EA.

To investigate the effects and mechanisms of EA in VH using a PI-IBS rat model, Sprague-Dawley rats were randomly divided into five groups: control group (n = 9); M group (model group, administered with TNBS, n = 9); M + EA group (administered with both TNBS and EA, n = 6); M + AP group (administered with both PAR2-AP and EA, n = 6); and M + EA + AP group (administered with TNBS, PAR2-AP, and EA, n = 6). TNBS was instilled into the anus of rats to induce the PI-IBS model, and VH was assayed by the abdominal withdrawal reflex (AWR) test. To examine whether EA alleviated VH via activated PAR2, the PAR2-AP, SLIGRL-NH2, was instilled into the anus of rats (once every three days, for a total of four treatments) four weeks post-TNBS enema. Control rats underwent the same procedure but were administrated intrarectally with saline solution. EA treatment at bilateral ST36 and bilateral BL25 were applied on rats after PAR2-AP enema (once daily for 15 min, five times a week for a total of two weeks). Control rats were fixed at the same time without any treatment. After each intervention, AWR tests were administered to evaluate VH. Finally, rat colon tissues were isolated to determine the protein and mRNA expression levels of PAR2, CGRP, SP, and TPSP by immunofluorescence, western blot, and real-time PCR. The experimental workflow is shown in Figure 1.

### 2.2. Animals

A total of 36 male Sprague-Dawley rats (180-220 g, aged 12-14 weeks) were obtained from the Experimental Animal Center of Nanjing University of Traditional Chinese Medicine, China. All animals were housed at an ambient temperature of 22°C and relative humidity of 40%–60% at the standard II animal house in the Experimental Animal Center with a light/dark cycle of 12 h/12 h. The feed adaptation lasted for seven days. All animal experimental procedures were performed according to the Guidelines for the Care and Use of Laboratory Animals (National Research Council, Washington, DC).

### 2.3. Model: PI-IBS VH Rats

The PI-IBS VH model was induced according to the internationally accepted method [13, 14]. Briefly, after 24 h of fasting, rats were deeply anesthetized with chloral hydrate (350 mg/kg, i.p.). While the rats were maintained in a head-down vertical position, a fine plastic catheter (external diameter = 0.96 mm) was gently inserted into the descending colon at a depth of 8 cm from the anus, and then TNBS (5 mg/rat, 0.8 mL in 50% ethanol) was instilled slowly into the colon lumen. After TNBS instillation, the catheter was left in place for 1 min and then slowly removed. The rats were left on a warm mound of bedding in head-down position to prevent drug leakage until they regained consciousness. Four weeks post-TNBS administration, visceral pain threshold pressure was measured. The rats with acquired VH (pain threshold pressure below 30 mmHg) were selected as the PI-IBS rats.

### 2.4. Assessment: Abdominal Withdrawal Reflex Test

The AWR test was performed as previously described to detect the pain threshold pressure. Briefly, rats were lightly anesthetized with ether to place a 6 cm long flexible latex balloon into the rectum through the anus. The end of the balloon was secured at least 1 cm proximal to the anal verge. Rats were then allowed to recover for at least 30 min. The tube of the balloon was connected via a connector to an injector and colorectal distension was applied in increments of 0.01 mL until a visible contraction of the abdominal wall was observed by an investigator blinded to the treatment. In this study, the pain threshold pressure was defined as the intensity of colorectal distension that elicited an observable reflex which was the lifting of the abdominal wall and body arching. The pain threshold pressure of all groups was recorded and repeated three times with intervals of at least 5 min for recovery.

### 2.5. Intervention: PAR2 Agonist Enema

Rats in the M+EA+PAR2-AP group and M+PAR2-AP groups received an enema of PAR2-AP which is an agonist of PAR2. Briefly, after 4 weeks of TNBS administration, the rats were lightly anesthetized with halothane. A fine plastic catheter (external diameter = 0.96 mm) was gently inserted into the descending colon at a depth of 8 cm from the anus. The rats were kept in a head-down vertical position, and then PAR2-AP [15] (0.8 mL SLIGRL-NH2 in 10% ethanol, 10% Tween 80, and 80% NaCl; 100 *μ*L per rat) was instilled slowly into the colon lumen. After PAR2-AP instillation, the catheter was left in place for 1 min and then slowly removed. The rats were left on a warm mound of bedding in head-down position to prevent drug leakage until they regained consciousness. Rats in other groups underwent the same procedure but were administrated intrarectally with saline solution. After 2 weeks PAR2-AP or saline solution administration (once every three days, four times total), EA treatment was applied.

### 2.6. Treatment: Electroacupuncture

Rats in the M + EA and M+ EA + PAR2-AP groups were fixed by special frames and treated with EA at bilateral ST 25 and ST 37, once daily for the first 5 days in a week as a single course, for a total of two courses. Stainless steel acupuncture needles (diameter: 0.3 mm) were inserted approximately 3 mm in depth with a dense-sparse waveform at a frequency of 2/15 Hz and retained for 15 min; the duration of two-frequency alternating is 3s. The needles were connected to a Han EA therapeutic stimulator (LH402A; Beijing Huawei Industrial Development Corporation, China). Rats in other groups were fixed by the same special frames at the same time but did not receive any treatment.

### 2.7. Sample Collection: Colon Tissues

After two weeks of EA treatment, the rats were sacrificed with urethane (25%, 1 mL/kg, i.p.) and tissues collected. Colon tissues were taken from the descending colon (12 cm above the anus), cleaned with normal saline, and then immediately frozen in liquid nitrogen. All samples were placed in a −80°C freezer and then transported to the Pathology Department of Jiangsu Province Hospital of Traditional Chinese Medicine for preservation.

### 2.8. Assessment: Immunofluorescence

For immunofluorescence analysis, all sections were blocked in 5% bovine serum albumin (BSA), followed by overnight incubation at 4°C with anti-PAR2 antibody (1:500; ab180953, Abcam, Cambridge, UK), anti-TPSP antibody (1:500; ab2308, Abcam), anti-CGRP antibody (1:500; ab47027, Abcam), or anti-SP antibody (1:500; Invitrogen, Carlsbad, CA, USA), then washed in phosphate-buffered saline (PBS) three times, and incubated at room temperature with fluorescein (FITC)-conjugated AffiniPure goat anti-rabbit secondary antibody (1:500; Zhongshan Gold Bridge, Beijing, China). Diaminobenzidine was used as the chromogen. 4′, 6-diamidino-2-phenylindole (DAPI) was added and incubated for 5 min at room temperature, and samples were viewed under a fluorescence microscope (Olympus-BH2, × 100 and × 400).

### 2.9. Assessment: Western Blotting

Proteins from colonic tissues were separated by sodium dodecyl sulfate-polyacrylamide gel electrophoresis and electrotransferred to PVDF membranes (TIANGEN, Beijing, China). Nonspecific binding sites were blocked using 5% skimmed milk in tris-buffered saline-Tween 20 (TBS-T) for 2 h at room temperature. The membranes were incubated at 4°C overnight with mouse anti-PAR2 antibody (1:1000; ab180953, Abcam), mouse anti-TPSP antibody (1:500; ab2378, Abcam), mouse anti-CGRP antibody (1:500; ab47027, Abcam), or mouse anti-SP antibody (1:500; Invitrogen). After washing with TBS-T three times, the membranes were subsequently incubated with anti-mouse secondary antibody (1:500; Zhongshan Gold Bridge) at room temperature for 1 h. As a protein loading control, all blots were stained with mouse anti-GADPH (1:10000; Zhongshan Gold Bridge). The bands were detected by an enhanced chemiluminescence technique (Amersham, Buckinghamshire, UK) on Kodak BioMax light film. Chemiluminescent signals of protein bands were quantified using Image J (National Institutes of Health, Bethesda, Maryland, USA).

### 2.10. Assessment: RT-PCR

Total RNAs were extracted from colon tissues of each group using Trizol reagent (JIALAN, Beijing, China). Subsequently, cDNA synthesis was performed with the Thermo Scientific RevertAid First cDNA Synthesis Kit (Thermo Fisher Scientific, USA). Quantitative real-time RT-PCR was performed using SYBR® Premix Ex Taq™ (Tli RNaseH Plus) on a LightCycler 480 system (A650t-1, Dalian, China). Samples were run in triplicate. The PCR amplification cycles were as follows: 30s at 95°C; amplification for 40 cycles, with denaturation for 10s at 95°C, annealing for 34s at 52°C, and extension for 34s at 60°C. Primers were as follows: TPSP-F (TGGCCATCAAGTACATGTTCG), TPSP-R (CGGTGCTCGGATGTAGAGCAGG), CGRP-F (CTGGAAGTTCTTTCCTTTTCTG), CGRP-R (GATCTCTTTGGGAAATGACAC), SP-F (ATGAAAATCCTCGTGGCGGT), SP-R (CAGCATCCCGTTTGCCCATT), PAR2-F (CACCACCTGTCACGATGTGCT), and PAR2-R (CCCGGGCTCAGTAGGAGGTTTTAACAC).

### 2.11. Statistical Analysis

Data were analyzed using SPSS 18.0 (SPSS Inc., Chicago, IL, USA) and plotted using GraphPad Prism 5.0 (GraphPad Software, CA, USA). All data are expressed as mean ± SEM (the standard error of the mean). Statistical comparisons for parametric data were carried out using one-way analysis of variance (ANOVA) followed by LSD tests. The statistical differences of nonparametric values among groups were identified using Kruskal-Wallis ANOVA followed by the rank-based Mann-Whitney U-test.* P *< 0.05 was considered statistically significant.

## 3. Results

### 3.1. EA Alleviates Visceral Hypersensitivity in a PI-IBS Rat Model

**Figure 2:**
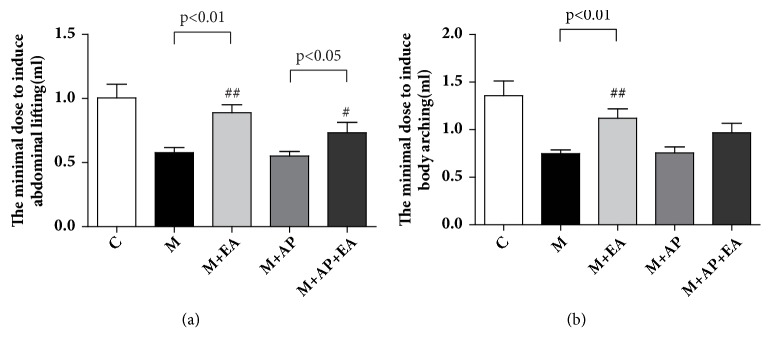
**EA alleviates VH in the IP-IBS rat model**. (a) The minimal dose required to induce the lifting of the abdominal wall in each of the five experimental groups. (b) The minimal dose required to induce body arching in each of the five experimental groups. Data are expressed as mean ± SEM (n = 9 rats for C and M group, n=6 for M+EA, M+AP, and M+EA+AP group), and statistical significance was determined using one-way ANOVA followed by Tukey's multiple comparison tests. The M group was administered with TNBS; the M + EA group was administered with both TNBS and EA; the M + AP group was administered with both TNBS and PAR2-AP; the M+AP+EA group was administered with TNBS, PAR2-AP, and EA.

To determine whether EA could regulate TNBS-induced VH, we compared the pain threshold levels, based on lifting of the abdomen and body arching, between the model (M) group and the M + EA group. As shown in Figure 2, compared with M group, a significant increase in the threshold to elicit lifting of the abdomen (Figure 2(a)) and body arching (Figure 2(b)) was observed in the M + EA group (lifting of the abdomen: 0.578 ± 0.115 versus 0.900 ± 0.201,* P *< 0.01; body arching: 0.748 ± 0.118 versus 1.100±0.316 in the M and M + EA groups, respectively,* P *< 0.01). A similar trend is observed between the M + AP group and M + AP + EA group, where there was a higher threshold for lifting of the abdomen and body arching in the M + AP + EA group compared with the M + AP group (lifting of the abdomen: 0.552 ± 0.107 versus 0.733 ± 0.214,* P *< 0.05; bodying arching: 0.756 ± 0.190 versus 0.967 ± 0.267 in the M + AP and M + AP + EA groups, respectively). To investigate the role of PAR2 in the effect of EA on VH, we treated rats with PAR2-AP, a PAR2 agonist. As shown in Figure 2, the threshold for lifting of the abdomen and body arching in the M + AP + EA group is lower than in the M + EA group, though the differences are not statistically significant (lifting of the abdomen: 0.733 ± 0.214 versus 0.900 ± 0.201,* P* > 0.05; body arching: 0.967 ± 0.267 versus 1.100 ± 0.316,* P *> 0.05).

### 3.2. EA Alleviates VH by Decreasing the Expression of Activated PAR2 in Colon Tissue

**Figure 3:**
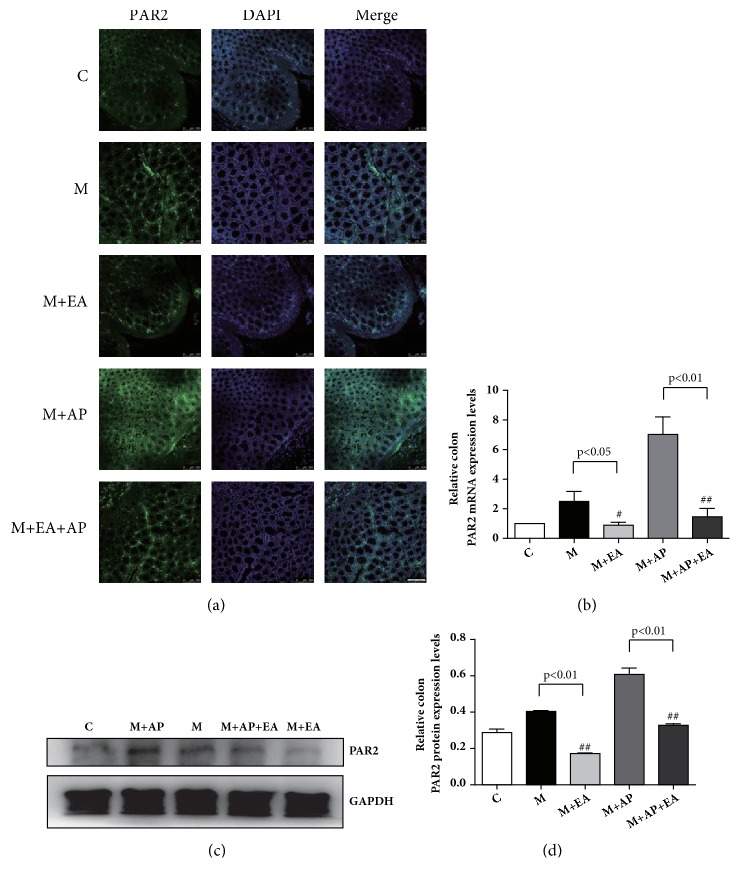
**EA alleviates VH through decreased expression of activated PAR2 in colon tissue**. (a) Confocal images of colon tissues isolated from rats in the five experimental groups and stained using anti-PAR2 (green); scale bar, 10 *μ*m. (b)* PAR2* mRNA expression as determined by RT-PCR. Expression was normalized to* GAPDH*. Each bar graph represents tissues pooled from three rats (n = 2 experiments for all five groups). (c) The western blot of PAR2 expression from colon tissues. (d) Quantification of PAR2 expression as determined by western blot, normalized to GAPDH expression. Each bar graph represents tissues pooled from three rats (n = 2 experiments for all five groups). Data in (b) and (d) were analyzed by one-way ANOVA followed by Tukey's multiple-comparison test. Data are expressed as mean ± SEM.

Immunofluorescence staining was used to assess the expression level of PAR2 in colon tissue. A typical IF image revealed that the expression level of PAR2 in the M group was significantly increased compared with the C group, and the M + EA group exhibited significantly decreased expression of PAR2 compared with the M group. Although the expression level of PAR2 in the M + AP + EA group was lower than that in the M + AP group, it was still higher than both the C group and the M + EA group (Figure 3(a)). RT-PCR analysis was performed to confirm the PAR2 mRNA expression levels, and western blot analysis was used to measure PAR2 protein expression levels. As shown in Figures 3(b) and 3(c), compared with the M group, the M + EA group displayed significantly decreased expression of both PAR2 mRNA and protein. A similar trend was observed between the M + AP group and the M + AP + EA group, with PAR2 protein and mRNA expression levels being obviously lower in M + AP + EA group than in the M + AP group. These results show that PAR2 protein and mRNA expression levels are higher in PI-IBS rats and that EA inhibits PAR2 activation.

### 3.3. EA Modifies the Abnormal Expression of TPSP in Colon Tissue

**Figure 4:**
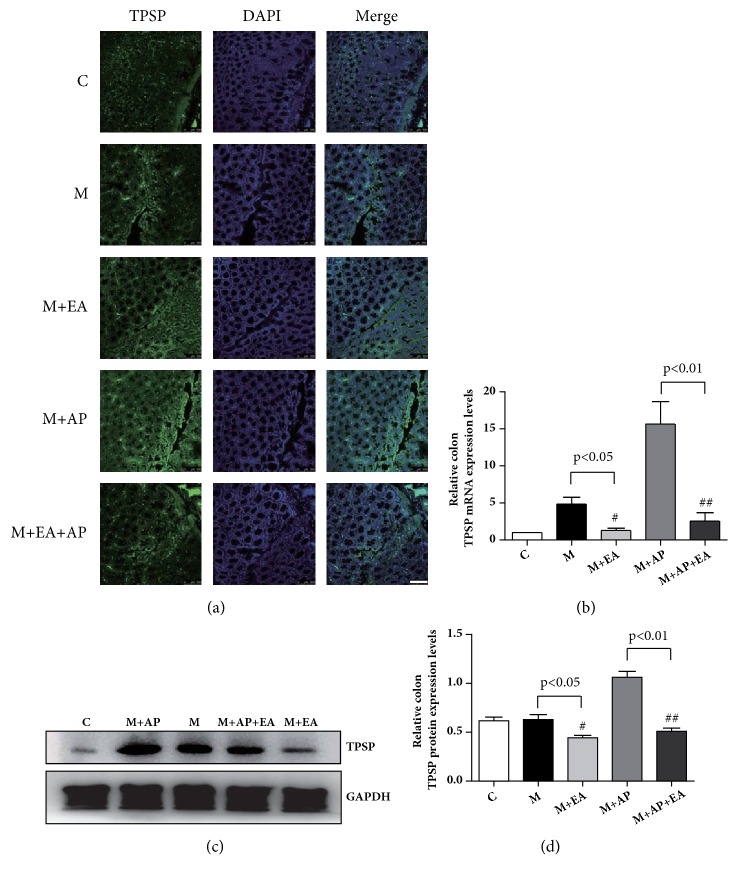
**EA alleviates VH through decreased expression of activated TPSP in colon tissue**. (a) Confocal images of colon tissues isolated from rats in the five experimental groups and stained using anti-TPSP (green); scale bar, 10 *μ*m. (b)* TPSP* mRNA expression as determined by RT-PCR. Expression was normalized to* GAPDH*. Each bar graph represents tissues pooled from three rats (n = 2 experiments for all five groups). (c) The western blot of TPSP expression from colon tissues. (d) Quantification of TPSP expression as determined by western blot, normalized to GAPDH expression. Each bar graph represents tissues pooled from three rats (n = 2 experiments for all five groups). Data in (b) and (d) were analyzed by one-way ANOVA followed by Tukey's multiple-comparison test. Data are expressed as mean ± SEM.

IF staining, western blotting, and RT-PCR were employed to assess the expression of TPSP, the crucial mediator of PAR2. IF staining showed that expression of TPSP was increased in the M group compared with the C group and the M + EA group (Figure 4(a)). As shown in Figures 4(b) and 4(c), compared with the M group, the M + EA group displayed significantly lower expression of both TPSP mRNA and protein. A similar trend was observed between M + AP group and M + AP + EA group, with the TPSP protein and mRNA expression levels lower in M + AP + EA group than in M + AP group. These results show that TPSP protein and mRNA expression levels are higher in PI-IBS rats and that EA downgrades the expression of TPSP.

### 3.4. EA Modifies the Abnormal Expression of CGRP in Colon Tissue

**Figure 5:**
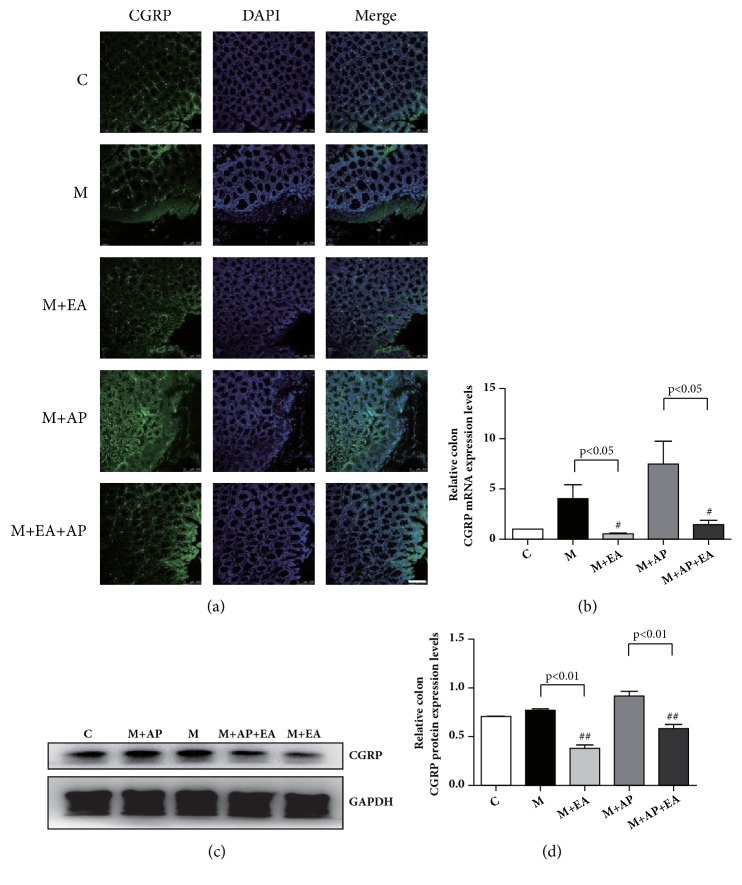
**EA alleviates VH through decreased expression of activated CGRP in colon tissue**. (a) Confocal images of colon tissues isolated from rats in the five experimental groups and stained using anti-CGRP (green); scale bar, 10 *μ*m. (b)* CGRP *mRNA expression as determined by RT-PCR. Expression was normalized to* GAPDH*. Each bar graph represents tissues pooled from three rats (n = 2 experiments for all five groups). (c) The western blot of CGRP expression from colon tissues. (d) Quantification of CGRP expression as determined by western blot, normalized to GAPDH expression. Each bar graph represents tissues pooled from three rats (n = 2 experiments for all five groups). Data in (b) and (d) were analyzed by one-way ANOVA followed by Tukey's multiple-comparison test. Data are expressed as mean ± SEM.

We next evaluated whether CGRP is affected by EA. As shown in Figures 5(a), 5(b), and 5(c), compared with the M group, the M + EA group exhibited significantly decreased expression of both CGRP mRNA and protein. A similar trend is observed in the M + AP group and the M + AP + EA group, with the CGRP protein and mRNA expression levels being lower in the M + AP + EA group than in the M + AP group. These results show that CGRP protein and mRNA expression levels are higher in PI-IBS rats than in controls and that EA inhibits CGRP activation.

### 3.5. EA Modifies the Abnormal Expression of SP in Colon Tissue

**Figure 6:**
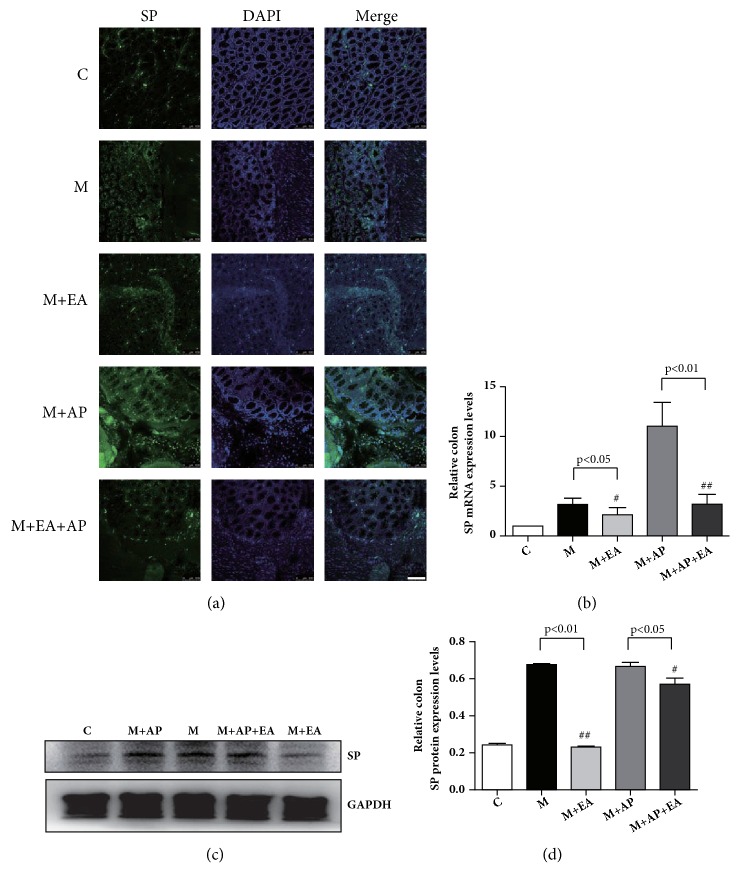
**EA alleviates VH through decreased expression of activated SP in colon tissue**. (a) Confocal images of colon tissues isolated from rats in the five experimental groups and stained using anti-SP (green); scale bar, 10 *μ*m. (b)* SP *mRNA expression as determined by RT-PCR. Expression was normalized to* GAPDH*. Each bar graph represents tissues pooled from three rats (n = 2 experiments for all five groups). (c) The western blot of SP expression from colon tissues. (d) Quantification of PAR2 expression as determined by western blot, normalized to GAPDH expression. Each bar graph represents tissues pooled from three rats (n = 2 experiments for all five groups). Data in (b) and (d) were analyzed by one-way ANOVA followed by Tukey's multiple-comparison test. Data are expressed as mean ± SEM.

SP is also an important downstream molecule for PAR2 activation. As shown in Figures 6(a), 6(b), and 6(c), compared with the M group, the M + EA group displayed significantly decreased expression of both SP mRNA and protein. A similar trend is observed in the M + AP group and the M + AP + EA group, with the SP protein and mRNA expression levels being lower in the M + AP + EA group than in the M + AP group. These results show that SP protein and mRNA expression levels are higher in PI-IBS rats than in controls and that EA inhibits SP activation.

## 4. Discussion

In this study, we investigated the role of PAR2 in EA-mediated alleviation of VH symptoms in PI-IBS rats. We used immunofluorescence, western blot, and RT-PCR analysis to determine the expression levels of PAR2, TPSP, CGRP, and SP in colon tissues. We analyzed differences between normal rats (C group), PI-IBS rats (M group), PI-IBS rats with EA administered (M + EA group), PI-IBS rats with PAR2-AP administered (M + AP group), and PI-IBS rats with PAR2-AP and EA administered (M + AP + EA group). Our results show that EA alleviates VH symptoms by downregulating the levels of PAR2 and TPSP, a protein upstream of PAR2, as well as the levels of the secondary neurotransmitters SP and CGRP in colon tissues in PI-IBS rats.

Consistent with previous reports [16–18], we found that EA can alleviate VH. Exploration of the underlying molecular mechanisms showed that PAR2 plays a critical role in the effects of EA in alleviating VH symptoms in PI-IBS rats. Based on this finding, we also investigated the upstream and downstream molecules of PAR2, including TPSP, CGRP, and SP. We found that EA decreases the protein and mRNA levels of PAR2, TPSP, CGRP, and SP. We also found that the effects of EA were reduced with the administration of the selective PAR2 agonist SLIGRL-NH2 (AP). Together, our results show that the PAR2 pathway plays a critical role in the effect of EA in alleviating VH symptoms in PI-IBS rats.

Recent studies have highlighted the importance of PAR2 in the pathogenesis of IBS [6, 19]. TPSP which is derived from degranulation of mast cells (MCs) is a ligand for PAR2. Interestingly, in this study, we did not observe a significant upregulation of TPSP in the model group (Figure 4(d)). PI-IBS model used in this experiment experienced a 28-day recovery phase. Therefore, mast cells may not be in the activation phase without external stimulation. This result suggests that it is not a simple increase of inflammatory mediators, but the upregulation or activity of PAR2 in nerve endings when inflammation occurs and its continuous neuroplastic changes, leading to excessive immune-neural reaction to normal stimulation which eventually produces visceral hypersensitivity. However, TPSP was observed to be significantly upregulated after the application of PAR2 agonists AP. This puzzling phenomenon may be explained through previous studies on the modulators of mast cells degranulation. Firstly, MC activation is affected by the CRF pathway which is one of the critical parts of the brain-gut axis [20, 21]. Previous study showed that CRF pathway activation in the central nervous system in TNBS-induced rat model of colitis which induced colonic hypersensitivity could be blocked by corticotropin-releasing hormone receptor 1 antagonist [22]. Therefore, AP-induced activation of pain ascending pathways may lead to the integration of central signals, affect the CRF signaling pathway, and then induce overactivation MC and upregulation of TPSP. Secondly, detailed data have shown that there are anatomical and functional interactions between MC and nerves in the intestine [23, 24]. Mast cells respond to neurotransmitters. Neuropeptides such as SP and CGRP activate human mast cell degranulation and chemokine production [25, 26]. Therefore, changes in neuronal excitation and plasticity induced by AP may induce activation of MC and upregulation of TPSP.

The analgesic effect of EA application in VH has been extensively reported [27–29], and therefore the molecular mechanisms involved in this effect are attracting more attention. Previous studies have indicated that the endogenous opioid pathway in the periphery [27, 28], the JAK2/STAT3 signaling pathway in the PAG-RVM-SCDH axis [29], the corticotropin-releasing hormone in the colon, spinal cord, and hypothalamus [15], and other pathways are all involved in mediating the effects of EA on VH. In agreement with previous research by our group [30], several studies indicate that EA attenuates VH via reduction of both colonic enterochromaffin cell number and 5-HT concentration in the brain-gut axis [29, 31–33]. Having identified that the effects of EA on IBS symptoms are closely related to 5-HT and CGRP, we went on to identify additional related inflammation signaling pathways.

Numerous studies have confirmed a critical role for PAR2 in inflammation, visceral- and cancer-evoked pain [10, 34], and recently several studies have provided evidence for a crucial role for PAR2 activated by TPSP in the colon tissues of IBS patients as a mediator of hypersensitivity symptoms [8, 9]. Additionally, activated PAR2 promotes the release of CGRP and SP in colon tissues and aggravates IBS symptoms, especially VH [34–38]. In our study, the abnormally high levels of PAR2, TPSP, CGRP, and SP in PI-IBS model rats could be altered with EA treatment. The administration of a selective PAR2 agonist, PAR2-AP, slightly decreased the effects of EA. The significant difference between these results may be explained in part by the fact that the TPSP/PAR2/SP/CGRP signaling axis in colon tissues participates in the analgesic effect of EA on VH in PI-IBS rats, and PAR2 may play the critical role in this signaling axis.

## 5. Conclusions

In conclusion, we have demonstrated the critical role of PAR2 in the analgesic effect of EA on VH in PI-IBS. We found that EA alleviates VH symptoms by downregulating the levels of the TPSP/PAR2/SP/CGRP signaling axis in colon tissues in PI-IBS rats. The major strength of this study lies in identifying the role of PAR2 in the mechanism of the EA effect. Our results add to the accumulating evidence that acupuncture regulates VH via inactivation of the TPSP/PAR2/SP/CGRP signaling axis. The next step is to explore whether EA relieves VH via this signaling axis in the central nervous system.

## Data Availability

The data used to support the findings of this study are available from the corresponding author upon request.

## References

[1] Thabane M., Kottachchi D. T., Marshall J. K. (2007). Systematic review and meta-analysis: The incidence and prognosis of post-infectious irritable bowel syndrome. *Alimentary Pharmacology & Therapeutics*.

[2] Schwille-Kiuntke J., Frick J.-S., Zanger P., Enck P. (2011). Post-infectious irritable bowel syndrome—a review of the literature. *Zeitschrift für Gastroenterologie*.

[3] Lee Y. Y., Annamalai C., Rao S. S. (2017). Post-Infectious Irritable Bowel Syndrome. *Current Fungal Infection Reports*.

[4] Long Y., Du L., Kim J. J. (2018). MLCK-mediated intestinal permeability promotes immune activation and visceral hypersensitivity in PI-IBS mice. *Neurogastroenterology & Motility*.

[5] Valdez-Morales E. E., Overington J., Guerrero-Alba R. (2013). Sensitization of peripheral sensory nerves by mediators from colonic biopsies of diarrhea-predominant irritable bowel syndrome patients: a role for PAR2. *American Journal of Gastroenterology*.

[6] Poole D. P., Amadesi S., Veldhuis N. A. (2013). Protease-activated receptor 2 (PAR_2_) protein and transient receptor potential vanilloid 4 (TRPV4) protein coupling is required for sustained inflammatory signaling. *The Journal of Biological Chemistry*.

[7] Zhao J.-M., Chen L., Zhou C.-L. (2016). Comparison of Electroacupuncture and Moxibustion for Relieving Visceral Hypersensitivity in Rats with Constipation-Predominant Irritable Bowel Syndrome. *Evidence-Based Complementary and Alternative Medicine*.

[8] Tian S.-L., Wang X.-Y., Ding G.-H. (2008). Repeated electro-acupuncture attenuates chronic visceral hypersensitivity and spinal cord NMDA receptor phosphorylation in a rat irritable bowel syndrome model. *Life Sciences*.

[9] Bueno L. (2008). Protease activated receptor 2: a new target for IBS treatment. *European Review for Medical and Pharmacological Sciences*.

[10] Cenac N. (2013). Protease-Activated Receptors as Therapeutic Targets in Visceral Pain. *Current Neuropharmacology*.

[11] Cenac N., Andrews C. N., Holzhausen M. (2007). Role for protease activity in visceral pain in irritable bowel syndrome. *The Journal of Clinical Investigation*.

[12] Qin H.-Y., Xiao H.-T., Wu J. C. Y., Berman B. M., Sung J. J. Y., Bian Z.-X. (2012). Key factors in developing the trinitrobenzene sulfonic acid-induced post-inflammatory irritable bowel syndrome model in rats. *World Journal of Gastroenterology*.

[13] Qin H.-Y., Xiao H.-T., Leung F.-P. (2012). JCM-16021, a Chinese Herbal Formula, Attenuated Visceral Hyperalgesia in TNBS-Induced Postinflammatory Irritable Bowel Syndrome through Reducing Colonic EC Cell Hyperplasia and Serotonin Availability in Rats. *Evidence-Based Complementary and Alternative Medicine*.

[14] Cenac N., Coelho A., Nguyen C. (2002). Induction of Intestinal Inflammation in Mouse by Activation of Proteinase-Activated Receptor-2. *The American Journal of Pathology*.

[15] Liu H.-R., Fang X.-Y., Wu H.-G. (2015). Effects of electroacupuncture on corticotropin-releasing hormone in rats with chronic visceral hypersensitivity. *World Journal of Gastroenterology*.

[16] Ma X. P., Tan L.-Y., Yang Y. (2009). Effect of electro-acupuncture on substance P, its receptor and corticotropin-releasing hormone in rats with irritable bowel syndrome. *World Journal of Gastroenterology*.

[17] Wu H.-G., Jiang B., Zhou E.-H. (2008). Regulatory mechanism of electroacupuncture in irritable bowel syndrome: preventing MC activation and decreasing SP VIP secretion. *Digestive Diseases and Sciences*.

[18] Jimenez-Vargas N. N., Pattison L. A., Zhao P. (2018). Protease-activated receptor-2 in endosomes signals persistent pain of irritable bowel syndrome. *Proceedings of the National Acadamy of Sciences of the United States of America*.

[19] Larauche M. (2012). Novel insights in the role of peripheral corticotropin-releasing factor and mast cells in stress-induced visceral hypersensitivity. *Neurogastroenterology & Motility*.

[20] Ayyadurai S., Gibson A. J., D’Costa S. (2017). Frontline science: corticotropin-releasing factor receptor subtype 1 is a critical modulator of mast cell degranulation and stress-induced pathophysiology. *Journal of Leukocyte Biology*.

[21] Saito-Nakaya K., Hasegawa R., Nagura Y., Ito H., Fukudo S. (2008). Corticotropin-releasing hormone receptor 1 antagonist blocks colonic hypersensitivity induced by a combination of inflammation and repetitive colorectal distension. *Neurogastroenterology & Motility*.

[22] Barbara G., Stanghellini V., De Giorgio R. (2004). Activated mast cells in proximity to colonic nerves correlate with abdominal pain in irritable bowel syndrome. *Gastroenterology*.

[23] Barbara G., Wang B., Stanghellini V. (2007). Mast cell-dependent excitation of visceral-nociceptive sensory neurons in irritable bowel syndrome. *Gastroenterology*.

[24] Kulka M., Sheen C. H., Tancowny B. P., Grammer L. C., Schleimer R. P. (2008). Neuropeptides activate human mast cell degranulation and chemokine production. *The Journal of Immunology*.

[25] Manning B. M., Gruba S. M., Meyer A. F., Haynes C. L. (2016). Neuropeptide-Induced Mast Cell Degranulation and Characterization of Signaling Modulation in Response to IgE Conditioning. *ACS Chemical Biology*.

[26] Zhou Y.-Y., Wanner N. J., Xiao Y. (2012). Electroacupuncture alleviates stress-induced visceral hypersensitivity through an opioid system in rats. *World Journal of Gastroenterology*.

[27] Xu G. Y., Winston J. H., Chen J. D. (2009). Electroacupuncture attenuates visceral hyperalgesia and inhibits the enhanced excitability of colon specific sensory neurons in a rat model of irritable bowel syndrome. *Neurogastroenterology & Motility*.

[28] Zhu X., Liu Z., Qin Y. (2018). Analgesic effects of electroacupuncture at ST25 and CV12 in a rat model of postinflammatory irritable bowel syndrome visceral pain. *Acupuncture in Medicine*.

[29] Wan J., Ding Y., Tahir A. H. (2017). Electroacupuncture attenuates visceral hypersensitivity by inhibiting JAK2/STAT3 signaling pathway in the descending pain modulation system. *Frontiers in Neuroscience*.

[30] Sun J.-H., Wu X.-L., Meng Y.-F. (2015). Electro-acupuncture decreases 5-HT, CGRP and increases NPY in the brain-gut axis in two rat models of Diarrhea-predominant irritable bowel syndrome(D-IBS). *BMC Complementary and Alternative Medicine*.

[31] Li H., Hu S., Zhang J. (2014). Effects and mechanisms of auricular electroacupuncture on visceral pain induced by colorectal distension in conscious rats. *Acupuncture in Medicine*.

[32] Chu D., Cheng P., Xiong H., Zhang J., Liu S., Hou X. (2011). Electroacupuncture at ST-36 relieves visceral hypersensitivity and decreases 5-HT3 receptor level in the colon in chronic visceral hypersensitivity rats. *International Journal of Colorectal Disease*.

[33] Farzaei M. H., Bahramsoltani R., Abdollahi M., Rahimi R. (2016). The role of visceral hypersensitivity in irritable bowel syndrome: Pharmacological targets and novel treatments. *Journal of Neurogastroenterology and Motility*.

[34] Zhong B., Wang D. H. (2009). Protease-activated receptor 2-mediated protection of myocardial ischemia-reperfusion injury: Role of transient receptor potential vanilloid receptors. *American Journal of Physiology-Regulatory, Integrative and Comparative Physiology*.

[35] Yoshida N., Kuroda M., Suzuki T. (2013). Role of nociceptors/neuropeptides in the pathogenesis of visceral hypersensitivity of nonerosive reflux disease. *Digestive Diseases and Sciences*.

[36] Berdún S., Rychter J., Vergara P. (2016). Surgical intestinal manipulation increases gene expression of TrkA, CGRP, and PAR-2 IN dorsal root ganglia in the rat. *Neurogastroenterology & Motility*.

[37] Bhatt D. K., Ploug K. B., Ramachandran R., Olesen J., Gupta S. (2010). Activation of PAR-2 elicits NO-dependent and CGRP-independent dilation of the dural artery. *Headache: The Journal of Head and Face Pain*.

[38] Liang W.-J., Zhang G., Luo H.-S., Liang L.-X., Huang D., Zhang F.-C. (2016). Tryptase and protease-activated receptor 2 expression levels in irritable bowel syndrome. *Gut and Liver*.

